# Neuroinflammatory Triangle Presenting Novel Pharmacological Targets for Ischemic Brain Injury

**DOI:** 10.3389/fimmu.2021.748663

**Published:** 2021-10-07

**Authors:** Zaib A. Shaheryar, Mahtab A. Khan, Ch. Sherjeel Adnan, Awais Ali Zaidi, Daniel Hänggi, Sajjad Muhammad

**Affiliations:** ^1^ Institute for Experimental and Clinical Pharmacology and Toxicology, University of Lübeck, Lübeck, Germany; ^2^ Faculty of Pharmacy, University of Lahore, Lahore, Pakistan; ^3^ Faculty of Pharmacy, University of Central Punjab, Lahore, Pakistan; ^4^ Imran Idrees College of Pharmacy, Lahore, Pakistan; ^5^ Department of Neurosurgery, University of Helsinki and Helsinki University Hospital, Helsinki, Finland; ^6^ Department of Neurosurgery, Faculty of Medicine and University Hospital Düsseldorf, Heinrich-Heine University Düsseldorf, Düsseldorf, Germany

**Keywords:** neuroinflammation, blood brain barrier, cytokine, brain microvascular endothelial cell, reactive oxidative species

## Abstract

Ischemic stroke is one of the leading causes of morbidity and mortality globally. Hundreds of clinical trials have proven ineffective in bringing forth a definitive and effective treatment for ischemic stroke, except a myopic class of thrombolytic drugs. That, too, has little to do with treating long-term post-stroke disabilities. These studies proposed diverse options to treat stroke, ranging from neurotropic interpolation to venting antioxidant activity, from blocking specific receptors to obstructing functional capacity of ion channels, and more recently the utilization of neuroprotective substances. However, state of the art knowledge suggests that more pragmatic focus in finding effective therapeutic remedy for stroke might be targeting intricate intracellular signaling pathways of the ‘neuroinflammatory triangle’: ROS burst, inflammatory cytokines, and BBB disruption. Experimental evidence reviewed here supports the notion that allowing neuroprotective mechanisms to advance, while limiting neuroinflammatory cascades, will help confine post-stroke damage and disabilities.

## Highlights

This review article highlights important cellular and subcellular targets for stroke, and might aid the scientific community in deeply understanding and targeting one or more these targets, to develop clinical therapeutic interventions in stroke.

## Introduction

Stroke follows heart diseases and cancer as the highest global cause of mortality. It is the leading cause of permanent disabilities (1). Stroke is generally classified into hemorrhagic and ischemic, with the latter involved in about 85% of stroke accidents (2). Hemorrhagic stroke underscores the rupture of intracranial aneurysm (ICA), dural arteriovenous fistula (dAVF), cerebral arteriovenous malformation (AVM), or rupture of small vessel due to hypertension, while ischemic stroke represents embolic or thrombotic occlusion in a brain artery (3, 4). In either of these cases, the repercussions are brain-tissue injury and functional disabilities due to damage to the respective brain region (5). The instant and primary damage to the brain cells are followed by neuroinflammatory cascade entailing bursts of reactive oxygen species (ROS), release of a variety of signaling cytokines, and damage to the cerebral microvasculature, as well as disruption of the blood-brain barrier (BBB) (6–9). These molecular neuroinflammatory mechanisms potentiate damage to the brain cells and influence the clinical outcome.

Neuroinflammation comprises of complex cellular and sub-cellular mechanisms triggered in response to injury in brain cells (10). The main driving factor for the carrying out of neuroinflammation is the bid to root out the damaging stimulus, however, once initiated it might become over activated, spreading initially to the damaged brain regions and initiating a range of intricate signaling pathways that advance the neuroinflammation to the next level (11, 12). In acute phase, the damaged neuronal cells and resident immune cells secrete inflammatory mediators including cytokines, free radicals, and prostaglandins to signal other inflammatory fragments (13). In response, neighboring glia and microglia are activated which further secrete chemical mediators to make arrangements for the disruption of the blood brain barrier and invite immune cells from systemic circulation to expand the scope of neuroinflammation (14). The ROS burst, cytokines, related subcellular pathways, and disrupted BBB all contribute to inflammation-mediated tissue damage (15). This triangle of ROS, cytokines, and BBB, is the most simplified concept in understanding how neuroinflammation advances brain injuries, particularly after stroke.

## Cytokine-Related Pathways in the Neuroinflammatory Triangle

Following ischemic stroke, damaged neurons, regional microglia, and astrocytes become the repository of initial stimuli for neuroinflammation. Microglial cells are a specialized population of macrophages that are resident in the brain and spinal cord (16). They do not inhabit a particular location, but rather advance to different areas in order to clear the debris of dead neurons, maintain tissue homeostasis, phagocytose infiltrating pathogens or necrotic cells, release immune factors that are either inflammatory or regulatory, and facilitate the repair process in damaged parts of the brain provoked by mediators released in the vicinity (17–21).

After the onset of ischemic stroke, it takes no longer than a few minutes to initiate the neuroinflammatory cascade (10). As soon as the microglial cells are activated, they not only change shape (morphological transformation) and polarize into special phenotypes, but also kickstart inflammatory signaling pathways (22).

Traditional approaches have classified activated brain microglia into two key sub-populations: M1 and M2 (23). To which phenotypic population they will switch to depends upon the nature and intensity of the stimuli (24, 25). M1 microglia are referred to as “pro-inflammatory” cells as they aggravate neuronal damage in ischemic brain regions along with disrupting the BBB to varying extents (26, 27). The compromised BBB subsequently allows infiltration of systemic leukocytes at the site of ischemic insult, expending the inflammatory cascade and neuronal damage (16). The reported pro-inflammatory mediators released and their respective pathways are discussed under separate headings below.

### Pro-Inflammatory Mediators Released by M1 Phenotype of Microglial Cells

Multiple proinflammatory cytokines are released by M1-type microglia that determine the tissue damage. Here, we review the most important cytokines released from the M1- type of microglia.

#### Tumor Necrosis Factor

TNF is a well-known proinflammatory cytokine that mediates tissue damage after stroke through multiple mechanisms. When TNF was administered directly to the brain parenchyma, it elicited local microvascular injury in the form of pericapillary edema (28) and leukocyte adhesion to cerebral capillaries (29). Similarly, when TNF was administered into the cerebroventricular space prior to ischemia, it augmented the extent of tissue damage, expanded the otherwise average infarct volume, and aggravated neurological deficits (30). These facts highlight the pro-inflammatory potential of TNF in brain ischemia. TNF mediates a neurodegenerative cascade through interferon receptors (INFRs) which are present on infiltrating macrophages, T-lymphocytes, glia, and neurons (31). TNF modulates gene expression *via* simple, direct signaling pathway resulting in secretions of pro-inflammatory mediators by these cells (32).

**Figure 1:**
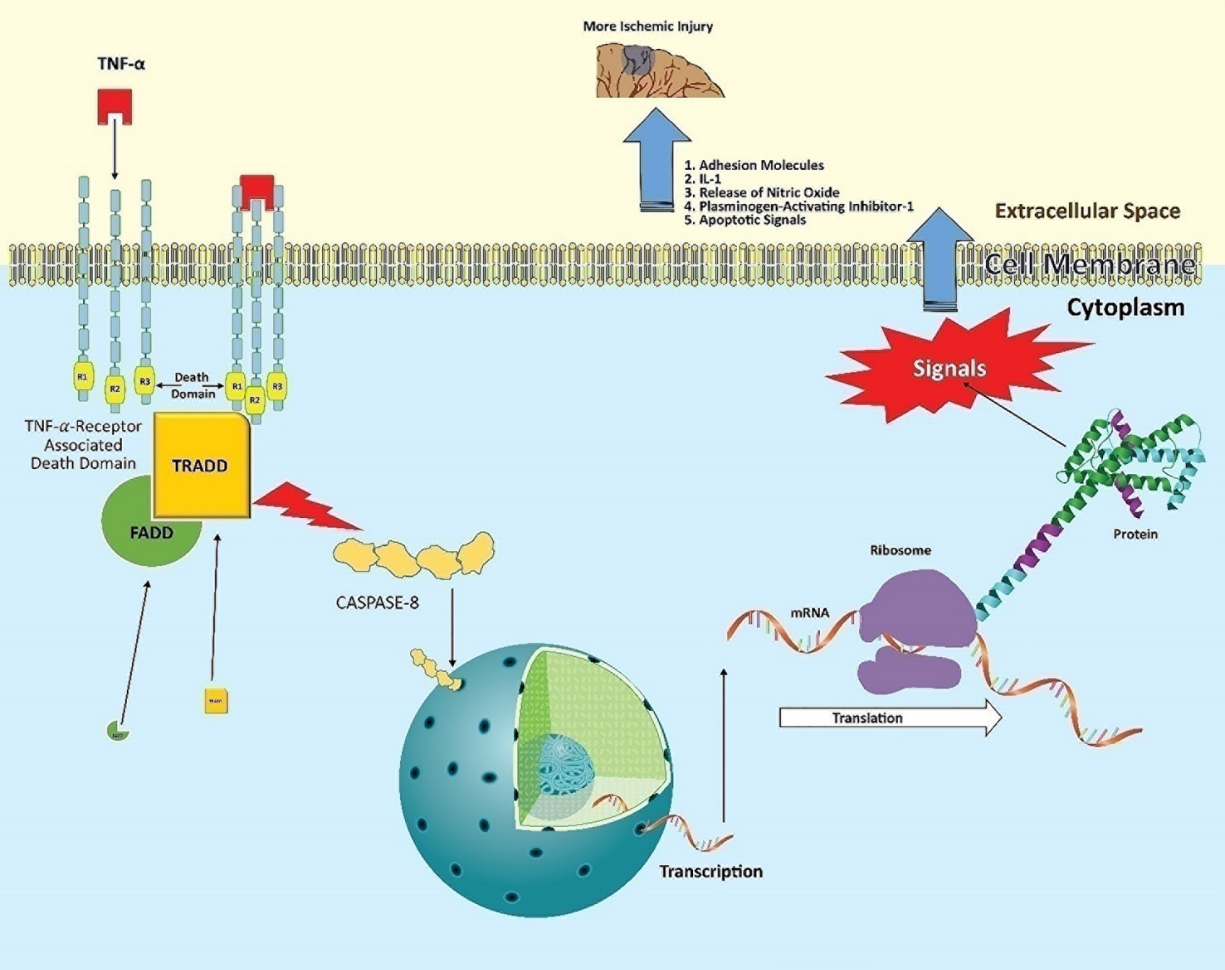
Post-stroke neuroinflammatory pathway of TNF. TNF released from activated microglia binds to the trimeric TNFR1, primarily present on infiltrating leukocytes. This allows the intracellular part of the receptor to complex with the adaptor proteins TRADD and FADD *via* its death domain. This domain activates cytoplasmic caspase-8 enzyme. Activated caspase-8 acts as a transcriptional factor, entering the nucleus to induce target genes. The expressed mRNA encodes proteins such as adhesion molecules, IL-1, nitric oxide synthase (nNOS), plasminogen-activating inhibitor-1, and apoptosis-inducing factors. This ‘mix’ contributes to the neuronal damage in ischemic tissue following stroke.

TNF released by activated microglia within the ischemic area of the brain binds to two types of receptors, namely TNF-receptors 1 (TNFR1) and TNF-receptor 2 (TNFR2) (33). The role of TNFR1 as well as its extended relationship with neurodegenerative outcomes have been well established. The activation of TNFR1 begins when the trimeric TNF binds to the trimeric receptor complex (**Figure 1**) (34). This triggers a complex formation with several adapter proteins, such as TNF-receptor-associated death domain (TRADD) and Fas-associated death domain (FADD). These adaptor proteins bind with the intracellular part of TNFR1, which consists of the 80-amino acid death domain (DD). The point at which the adaptor proteins bind is important (35).

We refer to this as the bifurcation point, as it will decide on the nature of signaling and further cell fate. Cell death signals will prevail if binding adapter proteins are Fas-associated protein and caspase-8 (that is probably the reason why caspase inhibitors reduce ischemic injury) (36). On the other hand, the inflammatory messages will be produced *via* expressing relevant genes when these adapter proteins are receptor-interacting protein kinase 1 (RIPK1) (leading to nuclear factor κB) or c-Jun N-terminal kinase (JUN) (37, 38). Consequently, infiltrating cells synthesize adhesion molecules and release IL-1, nitric oxide, and plasminogen activator inhibitor-1 (39). All these factors directly participate in neuronal degeneration and expanding stroke volume.

Apoptosis is a distinct form of cell death that is functionally and morphologically different from necrosis. Nuclear chromatin condensation, cytoplasmic shrinking, dilated endoplasmic reticulum, and membrane blebbing characterize apoptosis in general.

#### Interleukin-1β

Experimental studies show that the major form of IL-1 contributing to ischemic injury is IL-1*β* (40). In one study, chronic deletion of IL-1*β* receptors failed to deteriorate ischemic brain damage, employing the neurotoxic potentials of IL-1*β via* their receptors (41). IL-1 proteins are thus key players in signaling pathways such as apoptosis, toll-like receptors (TLRs), mitogen-activated protein kinase (MAPK), NOD-like receptors (NLRs), and nuclear factor κ-light-chain-enhancer of activated B Cells (NF-κB) (32).

The IL-1 family consists of eleven isoforms, out of which IL-1α and IL-1*β* play a strong pro-inflammatory role in neurodegenerative diseases (42). Another member of this family is the endogenous IL-1 receptor antagonist (IL-1Ra). In neurodegenerative diseases, IL-1*β* is considered one of the main culprits for infiltration of neutrophils, shattering of the BBB, astrogliosis, and neovascularization (43, 44). Support for this concept was obtained by employing the IL-1*β* antagonist on brain tissues. This significantly limited brain lesions as well as excitotoxic damage in rats (45).

**Figure 2:**
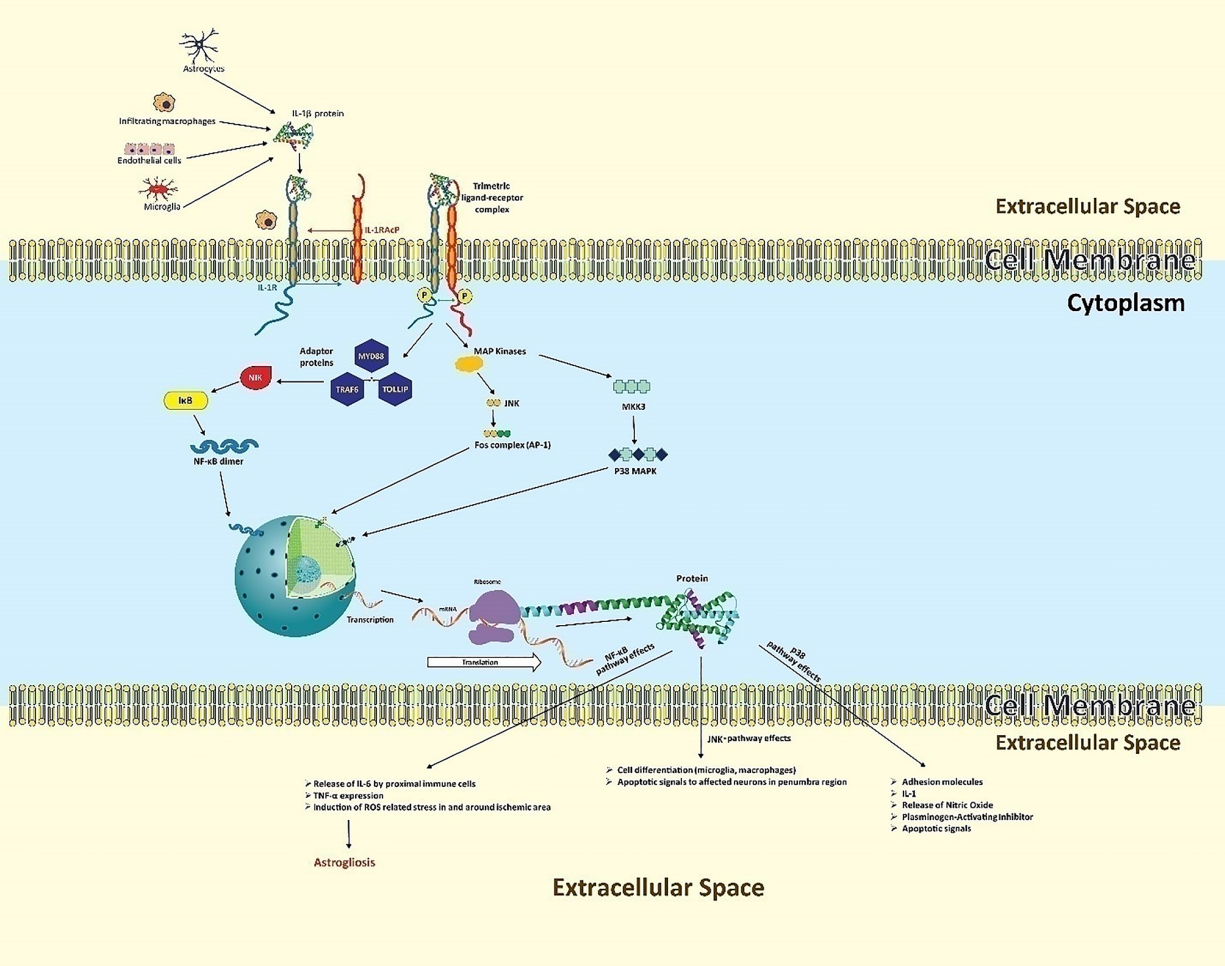
The neuroinflammatory cascades of IL-1*β* following ischemic insult. Secreted by microvascular endothelial cells, resident microglia, astrocytes, and infiltrating macrophages, IL-1*β* forms a complex with IL-1R1, aided by IL-1RAcP. This ligand-receptor complex triggers three key neuroinflammatory mechanisms. In the NF-κB pathway, ligand-receptor binding results in the cytoplasmic recruitment of three key adaptor proteins: MYD88, TOLLIP, and TRAF6. This leads to the activation of intermediate kinases, particularly NIK, which prevent IκB from inactivating NF-κB, indirectly allowing NF-κB-dimer formation. These dimers of NF-κB function as transcriptional factors, entering the nucleus to induce transcription of IL-1, TNF, and many other genes in various cell types. In the JNK pathway, downstream of the “death” signal, intracellular MAPKs phosphorylate c-Jun or related proteins, which heterodimerize with Fos proteins to form the transcriptional factor activator protein (AP-1). AP-1 regulates gene expression that mediates pro-inflammatory cellular processes, particularly proliferation and differentiation of infiltrating immune lineage as well as apoptosis of regional neurons. In the p38 MAPK pathway, ligand binding activates intracellular MKKK3 which in turn activates p38-MAPK, causing expression of diverse apoptotic signals, adhesion molecules for infiltration, and IL-1.

IL-1 produced by neurovascular endothelial cells, resident microglia, astrocytes, and infiltrating macrophages binds to IL-1 receptor type 1 (IL-1R1), localized in the cell membrane of all brain cells except microglia (46). Described in **Figure 2**, IL-1*β* is initially formed as a precursor protein, which is then cleaved and activated by caspase 1, followed by its release. The mature form of IL-1*β* forms a complex with transmembrane IL-1 receptor with the help of an accessory protein called IL-1 receptor accessory protein (IL-1RAcP) (47).The ligand-receptor complex triggers recruitment of various cytoplasmic proteins leading to multifarious intracellular signaling pathways guided by self-propagating cascades, namely deacetylation, phosphorylation, ubiquitination, methylation and palmitoylation, subsequently initiating three key neuroinflammatory mechanisms (48). The first key mechanism is the activation of NF-κB, second, the initiation of the c-Jun N-terminal kinases (JNKs) pathway, and third, the triggering of the p38 mitogen-activated protein kinase (P32 MAPKs) pathway (30, 49, 50).

In the first mechanism, NF-κB leads to the synthesis of neurotoxic or inflammatory mediators like TNF and diverse chemokines, collectively causing aggravated brain damage along with astrogliosis (51). NF-κB is a protein complex that controls gene expression in response to extracellular signals. This complex contains five structurally similar members: p50, p52, p65, c-Rel, and RelB (52). These proteins form NF-κB dimers to induce or, rarely, repress target genes (53). Depending on the ligand-receptor complex, various adaptor proteins are recruited and determine which type of message is to be transduced. For the activation of NF-κB, upstream signaling proteins include myeloid differentiation primary response 88 (MYD88), toll interacting protein (TOLLIP), and tumor necrosis factor receptor-associated factor 6 (TRAF6) (54). The recruitment cascade ultimately leads to activation of intermediate kinases such as interleukin-1 receptor-associated kinase (IRAK), receptor-interacting proteins (RIP), and NF-κB inducing kinase (NIK) (55, 56). In a normal biological state these intermediate kinases activate a biological complex called inhibitor of kappa B (IκB) (57), which inactivates NF-κB in cytoplasm to prevent gene expression. However, in a diseased or stress state (stroke), these IκB are phosphorylated, ubiquitinated, and then degraded. In the absence of IκBs, NF-κB subunits dimerize (e.g., p50 and p65) (49). The dimers translocate to the nucleus as a transcriptional complex.

In the second mechanism, the ligand-receptor complex activates c-Jun N-terminal kinases (JNKs) which exert the “death” signaling (58). The JNKs (JNK1 and JNK3) belong to the family of mitogen-activated protein kinases (MAPKs) that phosphorylate several proteins including the intracellular c-Jun protein which further heterodimerizes with Fos proteins (59). The complex of c-Jun and Fos protein is called transcriptional factor activator protein (AP-1) that regulates gene expression for pro-inflammatory cellular processes including proliferation and differentiation of infiltrating immune lineage as well as apoptosis of regional neurons (60–62).

In the third mechanism, the ligand-receptor complex activates mitogen-activated kinase kinase kinase-3 (MKKK3) which in turn activates p38-MAPK (63, 64).

The three transcription factors NF-κB, AP-1, and p38-MAPK help in modulating the gene expression for the desired biological response in the neuronal environment. The final outcomes of these expressions are manifested in the form of more inflammatory damage in the brain.

The intracellular and subcellular changes stimulate the expression of genes, which become key pro-inflammatory mechanisms. The JUN and p38 MAPK pathways, in parallel, induce the expression of genes for IL-6, IL-8, PCP1, COX-2, IL-1*β*, and MKP-1 (60).

The more IL-1*β* is expressed and released, the more other pro-inflammatory mechanisms will be activated.

#### Interferon Gamma

Apart from a variety of influential biological functions, IFN-γ (sourced by resident astrocytes as well as infiltrating T lymphocytes) up regulates the expression of class II major histocompatibility complex (MHC) molecules on the surface of macrophages and other T cells in brain tissues, which find their way through the disrupted BBB (65, 66). Immunohistochemistry and flow cytometric data show that activated CD4^+^ cells are found in and around the ischemic site with abundant MHC class II molecules on their surface for an effective inflammatory response (67, 68). Conversely, IFN-γ not only helps polarization of other distant microglial cells, but also influences the differentiation of CD4^+^, which further produces pro-inflammatory cytokines like IL-2, IFN-γ, and TNF (69, 70). Direct injection of IFN-γ into the rat CNS induced inflammation and cellular infiltration similar to that observed in chronic neurological diseases (71). This was indirectly evaluated by injecting IFN-γ in IFNGR-deficient mice (72). The results signified a lack of integrity and preservation of BBB.

In another mechanism, IFN-γ up-regulates the expression of IFN-α in microglial cells around the ischemic core, suppressing the neuroprotective role of microglia (73).

IFN-γ in the CNS facilitates helper T cell infiltration and neuroinflammation by inducing expression of vascular cell adhesion molecule 1 (VCAM-1), intercellular adhesion molecule 1 (ICAM-1), the chemokines CCL2 (recruits monocytes, memory T cells, and dendritic cells to the infarct area), CXCL9 (chemokine ligand 9 belonging to CXC chemokine family and induces chemotaxis, promotes differentiation and proliferation of leukocytes, and causes brain edema), and CXCL10 (secreted by infiltrating monocytes and endothelial cells in response to IFN-γ) (74–79).

Adding to the damage, IFN-γ increases the expression of MHC class II and co-stimulatory molecules on microglial cells (particularly M1 subtype), helping them act as antigen-presenting cells (APCs) for infiltrating myelin-specific T cells and leading to inflammation and demyelination (80, 81). One important thing to highlight here is the fact that increased concentrations of IFN-γ impairs the neuroprotective potential of M2 polarized microglial cells in severe inflammatory brain diseases (82).

By another mechanism, IFN-γ binds to INFRs located on neurovascular endothelial cells and immune cells to upregulate the expression of transmembrane intracellular adenosine molecule type-1(ICAM-1), resulting in infiltration of immune cells through the compromised BBB. IFN-γ also induces the gene expression of the vascular cell adhesion molecule (VCAM-1) on primary astrocytes, thereby further enhancing their role in inflammation (83, 84).

**Figure 3:**
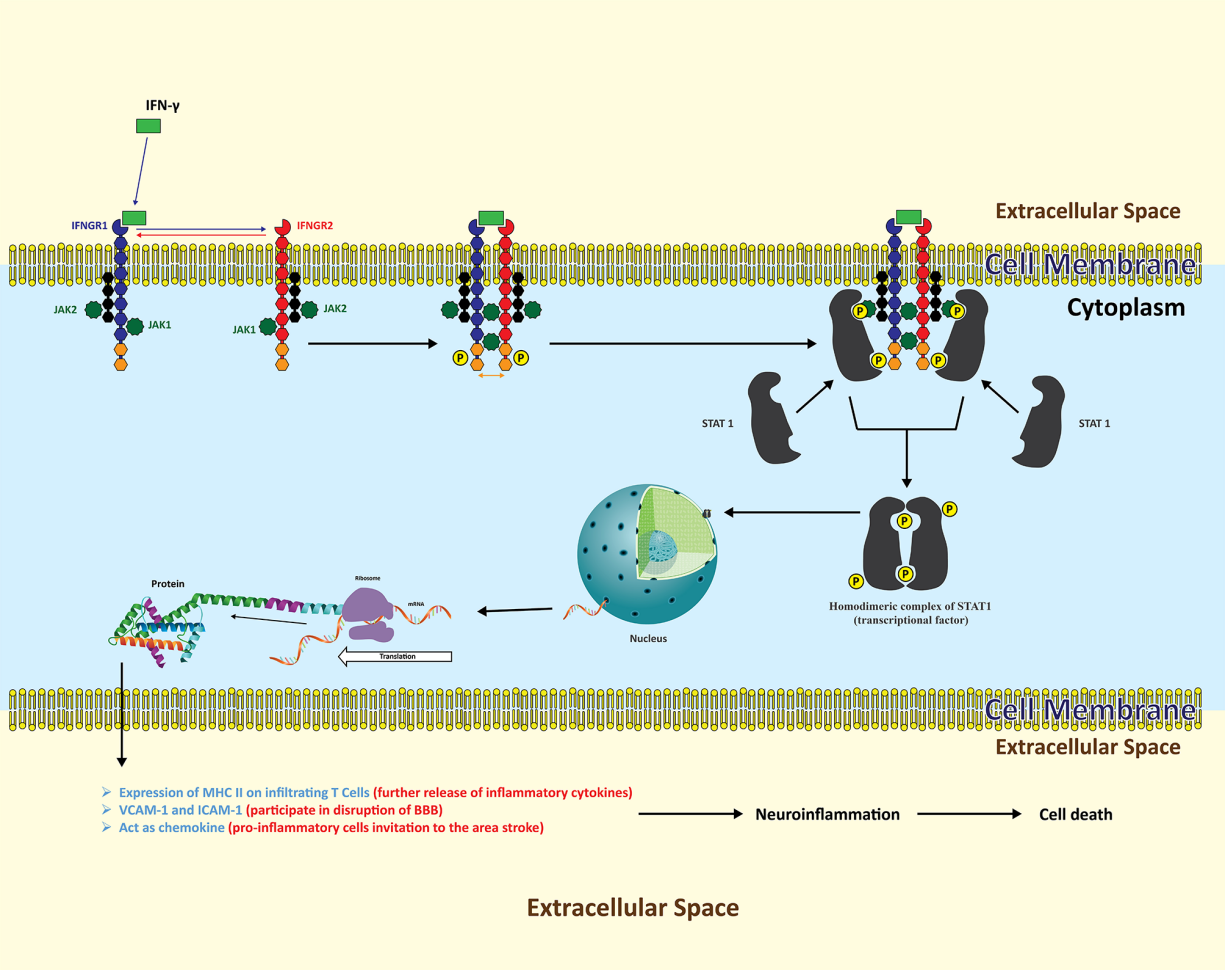
The neuroinflammatory cascades of INF-*γ* following ischemic insult. Resident astrocytes as well as infiltrating T lymphocytes are the key sources of INF-*γ*. Following release, INF-*γ* binds to transmembrane INFGR, which is further composed of two subunits, INFG1 and INFG2. Each receptor subunit of INFGR is further composed of two different proteins, with each bound to Jak1 and Jak2 respectively. INF-*γ* binding with INFG1 part brings INFG2 in to proximity with INFG1 in such a way that, intracellularly, Jak1 and Jak2 phosphorylate each other’s two receptor domains. These receptor domains now act as docking points for STATs to bind with respective JAKs. The activated STAT dissociates and forms a homodimeric complex functioning as a transcription factor inducing the expression of variety of protein signals in various cell types. In cerebral ischemia, upregulated proteins are adhesion molecules on a variety of cells that play their role in the disruption of the BBB and the infiltration of leukocytes, along with proliferation of pro-inflammatory cells.

IFN-γ acts by binding and signaling *via* interferon gamma receptors (INFGR), which are overly expressed during inflammatory conditions in the brain (**Figure 3**) (85). IFNGR1 and INFGR2 consist of two different protein subunits (86). Both receptors are not in proximity to each other until IFN-γ binds to the extracellular domain of INFGR1 and triggers the cascade (87). Inside the cell, each subunit of IFNGR1 and INFGR2 is attached to a unique member of the Janus family kinase family (Jaks), i.e., Jak1 and Jak2, respectively (88). As soon as IFN-γ binds to the extracellular domain of IFNGR1, it brings IFNGR1 and INFGR2 in close proximity, clearing the way for the point where IFN-γ binds with IFNGR1 and INFGR2. Subsequently, Jak1 and Jak2 phosphorylate each other on each receptor domain. Phosphorylated sites become docking points for an intracellular transducer and activator of transcription (STATs). This will result in phosphorylation of STATs by respective JAKs. The activated STAT1 dissociates and forms a homodimeric complex and travels to the nucleus where it binds to its genes to induce transcription (89, 90).

By such signaling, IFN-γ participates in modulating gene expression in microglia and astrocytes (91). Elevated levels of MHC II and ICAM-1 (intracellular adhesion molecule type 1) on the surface of these cells transform the pathophysiological environment suitable for infiltration of leukocytes, release of inflammatory cytokines, and enlargement of stroke lesions (92).

Despite various new studies exploring interactive effects of IFN-γ, it is becoming evident that this cytokine not only has protective effects but also well proven proinflammatory and deleterious consequences in the brain (89, 93).

#### Interleukin 6

A major proinflammatory cytokine which is implicated in the expansion of ischemic volume is interleukin 6 (IL-6) (94). Diverse stroke studies underscored the significant increase in the levels of IL-6 immediately after stroke onset (95, 96). Many of these studies implicate IL-6 as a key contributing factor in the pathogenesis of ischemic stroke, however, some of the research studies faintly underscore the contrary. Taking the convincing evidence, IL-6 is being discussed here as a neuroinflammatory protein.

Resident microglia, endothelial cells of the cerebral circulatory system, and infiltrating macrophages and T cells are fundamental sources of its release (97–99). It acts as a messenger protein between leukocytes, endothelial cells, and parenchymal cells (100). The pathophysiological manifestations produced in response to IL-6-related signaling mechanisms are multifaceted and complex. However, various studies have pointed to some key effects of IL-6: proliferation of infiltrating immune cells, expression of genes related to growth inhibitory proteins, and apoptosis-inducing endogenous secretions (28, 101, 102). These conclusively add more to the neuroinflammation and its deteriorating effects.

**Figure 4:**
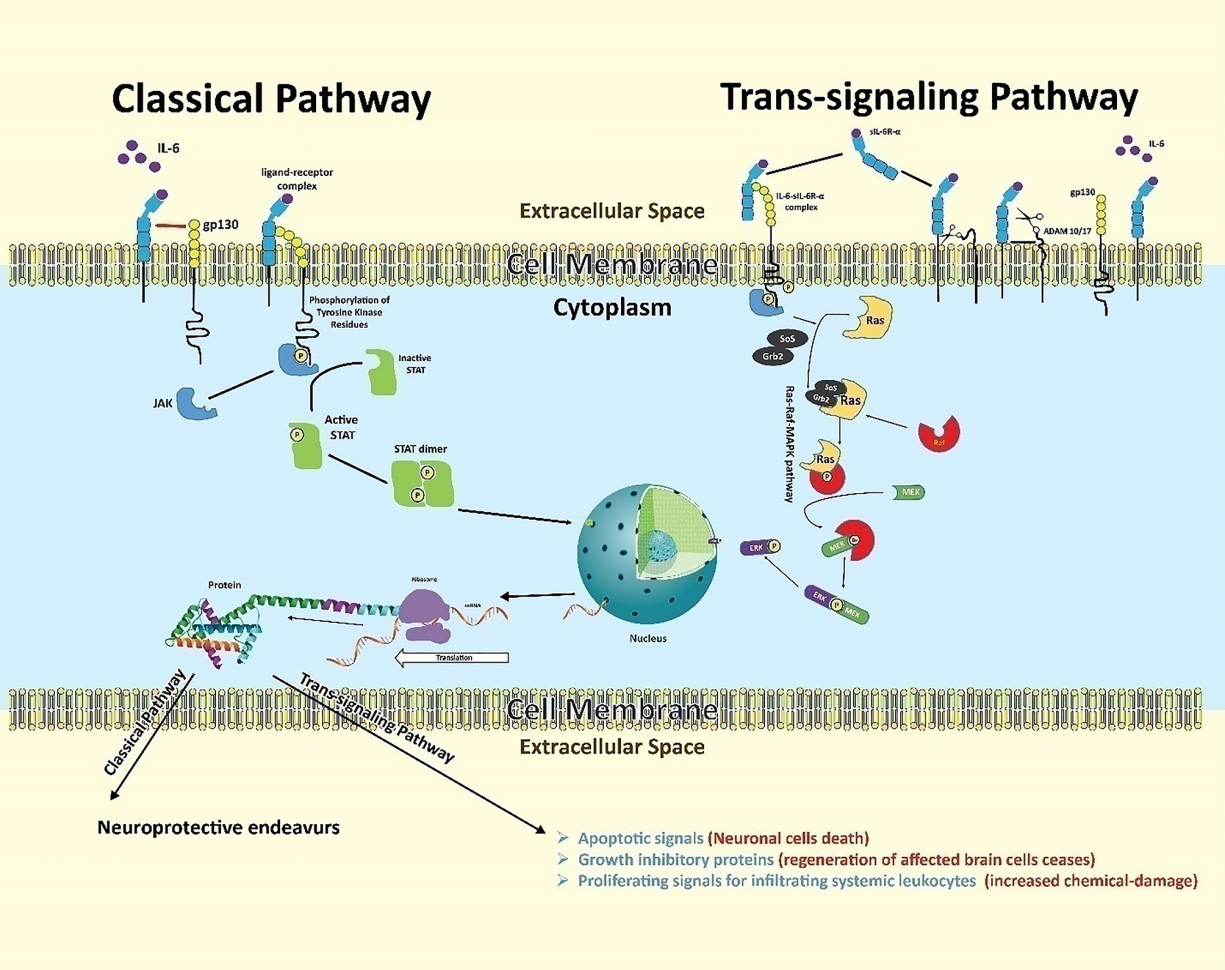
Neuroinflammation downstream of IL-6 in stroke. IL-6 binds its receptor and proceeds two separate but related pathways: the classical and trans-signaling pathways. The classical pathway begins when IL-6 binds with IL-6R-α chain subunit assisted by gp130, an intra-cellular signal transducer. This binding allows intracellular, receptor-associated JAKs to phosphorylate each other on respective tyrosine domains. Phosphorylated JAKs further phosphorylate related tyrosine residues on the receptor, creating binding sites for proteins possessing SH2 domains. Cytoplasmic STATs then bind to these SH2 domains to be phosphorylated by respective JAKs, making them dissociate from the receptor. The dissociated STATs form dimers, which act as transcription factors. The dimers translocate to the cell nucleus to induce gene expression for subsequent protein signaling outcomes. STATs may also be tyrosine-phosphorylated directly by receptor tyrosine kinases - but since most receptors lack built-in kinase activity, JAKs are usually required for signaling. The trans-signaling pathway begins when IL-6 ligands with sIL-6R-α in the extracellular matrix. The high affinity drives this complex to bind with gp130 expressed overly on glial and neuronal cells following ischemic insult. This trio-binding results in the activation of intracellular tyrosine-associated JAKs. Phosphorylated tyrosine-sited allow the adaptor proteins (SoS and Grb-2) to bind and convert inactive cytoplasmic RAS to an active one, initiating a series of phosphorylations of a variety of molecules like Raf, MEK, and ERK. The activated ERK acts as a transcriptional factor. It enters the nucleus to express targeted genes for inflammatory protein signaling.

IL-6 signaling transduction is mediated by the IL-6 receptor (IL-6R) found on a limited subset of immune cells in the brain including microglia, endothelial cells, and infiltrating T cells (not on oligodendrocytes and astrocytes) (103). The IL-6 receptor is activated *via* two separate, but related, pathways (**Figure 4**), the classical and the trans-signaling pathways (104).

The classical pathway (producing neuroprotective outcomes) begins when IL-6 binds with membrane bound IL-6R. The membrane-bound IL-6R is composed of two subunits: the IL-6R-α chain (to which IL-6 binds) and the assisting transmembrane signaling subunit, glycoprotein 130 (gp130), which is the intra-cellular signal transducer and abundantly expressed during ischemic stroke (105, 106). This gp130-associated signaling utilizes intracellular JAK/STAT signaling pathways (107). In this pathway, the complex of IL-6, IL-6R-α, and gp130 results in bringing two receptor-associated JAKs (one on each intracellular receptor domain) into close proximity. Each JAK is phosphorylated by the other on a respective tyrosine domain. The activated JAKs in turn phosphorylate related tyrosine residues on the receptor, creating binding sites for proteins possessing SH2 domains (5). STATs bind to the phosphorylated tyrosines on the receptor using their SH2 domains, and are then tyrosine-phosphorylated by JAKs, causing the STATs to dissociate from the receptor (2). These activated STATs form hetero- or homodimers, where the SH2 domain of each STAT binds the phosphorylated tyrosine of the opposite STAT, and the dimer then translocates to the cell nucleus to induce transcription of target genes (2). STATs may also be tyrosine- phosphorylated directly by receptor tyrosine kinases; however, since most receptors lack built-in kinase activity, JAKs are usually required for signaling.

In trans-signaling pathway (promote inflammatory outcomes), many cell types in the brain respond to released IL-6 by releasing a soluble form of IL-6R-α (sIL-6R-α) in the extracellular environment. Enzymes cut free the sIL-6R-α at the basepoint near the cell membrane (108–110).. This cleavage is done by metalloproteases including A Disintegrin And Metalloproteinase (ADAM) family members ADAM10 and ADAM17 (111). Shedding of the sIL-6R-α allows the free receptor to bind with IL-6 ligand in the extracellular matrix. This sIL-6R-α and IL-6 complex has a very high affinity to membrane bound gp130 (present on the cell membrane of distant glial and neuronal cells), causing activation of intracellular tyrosin-kinases, such as Janus kinase (JAK), which in-turn activates two pathways: activation of JAK-STAT pathway (upregulate the synthesis of iNOS, T cell differentiation)and the RAS-RAF-MAPK pathway (112–114).

Both these mechanisms lead to expression of genes which are associated with producing inflammatory outcomes (115). IL-6 also induces excess production of vascular endothelial growth factor (VEGF), leading to enhanced vascular permeability, which is one of the many pathological features of inflammatory lesions in the brain (116).

## ROS Burst in Neuroinflammatory Triangle

Reactive oxygen species (ROS) are a group of reactive oxygen-containing molecules including superoxide, peroxides, single oxygen, and hydroxyl radical (117, 118). ROS perform significant physiological roles in diverse biological processes such as intracellular signaling, regulation of transcription, immune response modulation, and apoptosis (119–122).

Under normal physiological conditions in the brain, ROS are produced through two pathways: enzymatic and non-enzymatic pathways (123). Enzymatic pathway is undertaken intracellularly by endogenous enzymes, while non-enzymatic pathway is carried out *via* antioxidative mechanisms (124, 125). Brain cells entail enzymes such as superoxide dismutase (SOD), glutathione peroxidase (GPX), and catalases (CAT) (126). SOD primarily dismutases superoxide to hydrogen peroxide which is further broken down into water and oxygen by GPX and CAT (127). In the second pathway, ROS level in the extracellular environment is regulated exclusively by small antioxidant molecules which can be water-soluble or lipid-soluble (128). These antioxidant molecules include Vitamin C, Vitamin E and glutathione, N-acetylcysteine, and melatonin (129, 130). Normally, equation of ROS generation is balanced by the neutralization of ROS by antioxidants (131). Functional roles of ROS at cellular and subcellular levels are unique and diverse.

ROS are central to apoptosis, which in turn is key to maintaining cellular homeostasis like synaptic activity, maintenance of vascular tone, and mediation of inflammatory response (132–134). Apoptotic signaling is translated by ROS through the activation of c-Jun N-terminal kinases and activation of death receptors (135). Similarly, immune cells (microglia and infiltrating macrophages) in the brain are only able to completely digest the engulfed microbes through intracellular oxidative burst (121, 136). Also, a variety of cellular signaling in the brain requires ROS for the activation of transcriptional factors like p53 and NF-κB for functional outcomes. ROS have a central role to play as second messengers for epidermal growth factor, platelet-derived growth factor (137), and substance P receptor (138–141).

In the post-stroke phase, the damaged neurons give rise to ROS burst when anti-oxidants fail to maintain this equation resulting in excessive oxidative stress (121). Resultantly, a series of chemical reactions begin where ROS reacts with cellular components of brain cells (142). The more brain cells die, the more the neuroinflammatory effects are.

**Figure 5:**
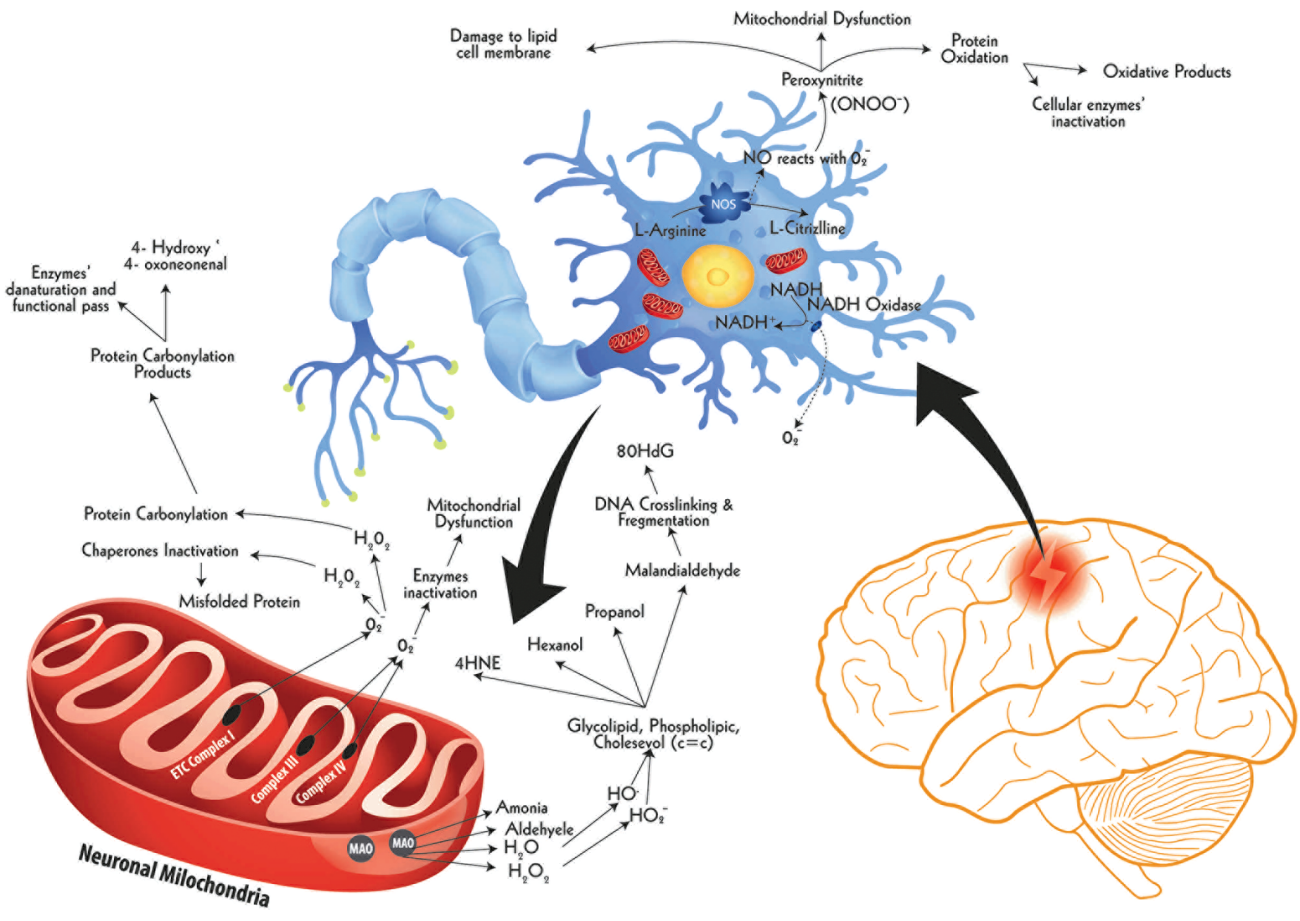
The ROS burst following ischemic insult and damage to biological molecules. Following hypoxia, brain cells witness a swift imbalance between ROS production and their neutralization mechanisms, particularly in neuronal cells, resulting in a constant rise in ROS levels, called ROS burst. One of the key sources of ROS burst inside the cell is mitochondria, others being cell membrane and peroxisomes. These ROS molecules damage cellular parts *via* protein oxidation, lipid peroxidation, and DNA damage. The neuronal nitric oxide synthase (nNOS) in the cytoplasm of hypoxic neuron produces excessive production of Nitric Oxide (NO) which after release reacts with cellular oxygen producing peroxinitrite (ONOO⁻), a powerful oxidant damaging cytoplasmic proteins and lipid composites. Another ROS-releasing source during hypoxia is the extracellular space-facing membrane bound enzyme called NADH oxidase. This enzyme releases ample quantities of superoxide molecules with respective damaging implications. A third major source of ROS release is the mitochondria. The external membrane-bound monoamine oxidase (MAO) releases a range of reactive molecules, primarily H2O2, which causes the production of hydroxyl radical (HO•) and hydroperoxyl radical (HO2⁻). These two radicals cause lipid peroxidation by selectively attacking carbon-carbon double bond (c=c) of saturated lipid compounds, releasing by-products such as 4-Hydroxynonenal (4-HNE), Hexanol, Propanol, and Malondialdehyde. Of these, the latter is a highly reactive organic compound, aggressively reacting nucleic material to cause DNA fragmentation. The key DNA fragmentation marker of this pathway is 8-Hydroxydeoxyguanosine (80HdG). The hypoxia-induced impaired mitochondrial functions, especially affected Complex I, III, and IV, which drive Electron Transport Chain (ETC), release abundant quantities of Superoxides (O2•⁻) into the cytoplasm. These superoxides further produce reactive species which have two-fold damaging effects. On the one hand, these inactivate chaperones result in increased levels of misfolded proteins and, on the other hand, cause carbonylation of cellular proteins. This carbonylation of proteins is evident by the elevated levels of carbonylation marker in stroke, such as 4-Hydroxy, 4-oxoneonenal.

In a stroke episode, ROS burst causes generalized protein oxidation by disrupting peptide bonds in the amino acid chain, resulting in cross-linkage and denaturation of proteins (**Figure 5**) (143). Structural alteration in protein molecules causes enzyme inactivation (119) and ion-channel dysfunction (144). One study has demonstrated how protein oxidation causes neurotoxicity by triggering denaturation of glutamine synthetase in mice. In astrocytes, glutamine synthetase transform glutamate to glutamine to protect the neuronal construct against excitotoxicity (145). However, following ischemic stroke, denaturation of various cerebral enzymes fans neuroinflammatory outcomes (27). Lipid peroxidation is another feature of ROS in brain tissues (146).

**Figure 6:**
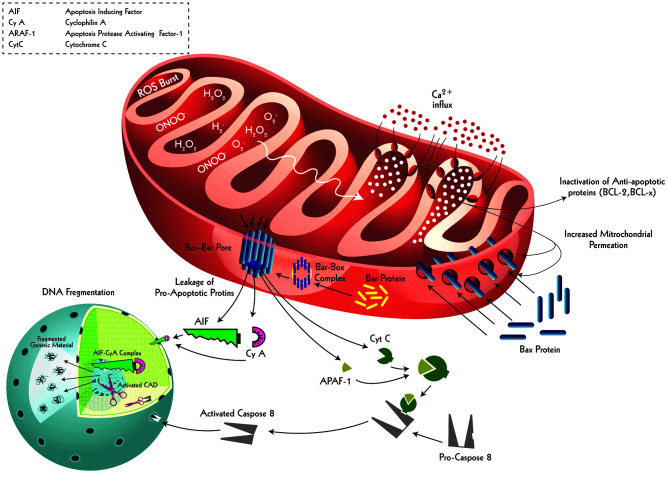
Intrinsic apoptotic signaling and resultant neuronal death driven by ischemic-induced ROS burst. Ischemic events cause mitochondrial matrix of the neurons uptake extracellular calcium as a result of augmented mitochondrial respiratory burst. This calcium accumulation not only causes inactivation of anti-apoptotic proteins (BCL-2 and BCL-x) inside mitochondria but also results in an increase in the permeability of the outer and inner mitochondrial membranes. This permeation allows cytoplasmic Bax proteins to enter and make ‘tunnel-like’ complexes with mitochondrial Bar proteins, which then embed into the outer mitochondrial membrane as Bax-Bar pores. These specialized pores serve as channels to transport intra-mitochondrial pro-apoptotic proteins (AIF, Cy A, APAF-1, and Cyt C) into the cytoplasm. The AIF and Cy A make a complex (AIF-Cy A complex), directly entering the nucleus to inflict defragmentation. Similarly, APAF-1 binds with Cyt C to transform pro-Caspase 8 protein into an active form: Caspase 8. The Caspase 8 enters through the nuclear pores to activate Caspase Activated DNase (CAD) which defragments the nuclear material, a key apoptotic signaling outcome. This dual apoptotic signaling effect is primarily initiated by a respiratory burst in the affected neuron following ischemic insult.

The phospholipid cell membrane of brain cells is concentrated with fatty acids, particularly polyunsaturated fatty acids. Lipid structures have carbon-carbon double bonds (C=C) which are the target sites for ROS (147). ROS interaction with C=C in lipid molecules produces lipid radicals which further interact with oxygen to make lipid peroxyl radical (148).This lipid peroxyl radical reacts with another proximally available lipid acid to synthesize lipid radical and lipid peroxide (149). These two lipid radicals give rise to highly reactive malondialdehyde (MDA) and (150) (4-HNE), the end products and markers of lipid peroxidation (151). MDA and 4-HNE have devastating effects on neighboring neuronal and non-neuronal cells (152). MDA reacts with enzymatic proteins to form advanced end-products of lipid peroxidation, further contributing secondary deleterious effects in neuroinflammation (153). Parallel to this, 4-HNE chemically reacts with hydroxyl group, aldehyde, and C=C in various biological molecular structures to paralyze them functionally (150). It has a direct role to play in advancing neuroinflammatory cascade by acting as a second messenger in the regulation of various transcriptional factors such as erythroid 2-related factor 2, activating protein-1, NF-κB, and peroxisome-proliferator-activated receptors (154). Various studies have also highlighted the influential role of 4-HNE in MAPK and PI3K/AKT pathways to produce damaging effects following ischemic stroke (155). In one study, vitamin E has demonstrated reduced lesion volume and diminished behavioral impairments in animal MCAO models (156). Also, EPC-K1, a Vitamin C analogue, reduced lesion size in rat MCAO model by limiting lipid peroxidation (157). Mitochondrial respiratory burst is another major contributory factor in neuronal cell death following stroke, saliently explained in **Figure 6** here.

Increased concentration of 8-hydroxy-2’-deoxyguanosine (8OHdG) is a fundamental marker used by various studies suggesting oxidative DNA damage (158). In the ischemic brain, DNA oxidation suggests DNA disintegration, crosslinking of DNA with denatured protein molecules, as well as DNA mutation (159).

Oxidative stress significantly contributes to the detrimental effects of neuroinflammation. Various antioxidant therapeutic approaches, i.e. polyethylene glycol-conjugated SOD (PEG-SOD) (160) and polyethylene glycol-conjugated CAT (PEG-CAT) (161), underscore that timely interventions following stroke attenuate neuroinflammatory damage partially due to limiting the ROS generation (162).

## Blood-Brain Barrier in Neuroinflammatory Triangle

The integrity of neurovascular structure is crucial to maintaining a neurophysiological barrier against movement of ions, molecules, systemic immune cells, and subcellular components (163). This barrier is called the Blood Brain Barrier (BBB) and is composed of four key components: brain microvascular endothelial cells (BMEC), Astrocytes, Pericytes, and Microglial cells (164).

BMEC have contrasting features to systemic endothelial cells with closely-fitted junctions to give high ionic transportation, paracellular flux, presence of disproportionately distribution of enzymes, and hermetically sealed carrier-mediated transport system (165). A variety of biological compounds (from glucose to amino acids, and from exogenous drugs to minerals) enter the brain tissue *via* a special carrier-protein system which is abundantly expressed in BMECs (166). Apart from expressing brain-derived neurotrophic factor (BDNF), transferrin receptor proteins, insulin receptors, and insulin-like growth factor receptor, BMECs also express powerful vasoactive endothelin-1 (ET-1; the other less common isoforms are ET-2 and ET-3) and vasodilatory nitric oxide (NO) (167–171).

A balance between ET-1 and NO is crucial for ensuring normal homeostasis in the brain (172). A sudden change in this balance (ischemic stroke) inflicts pathophysiological devastation (173). Expression of ET-1increases in response to ROS burst, neuronal damage, inflammatory cytokines, and thrombin (174).Within a day, the level of ET-1 in Cerebrospinal Fluid (CSF) rises significantly due to astrocytes and endothelial cells which bind numerously expressed ET-1 receptors localized on neurons, glial cells, microvascular endothelial, and smooth muscle cells to give rise to assorted paracrine physiological effects (175–179).

Firstly, ET-1 upsurges the expression of a variety of adhesion molecules from BMECs such as intercellular adhesion molecule 1 (ICAM-1; CD54) (180), vascular cell adhesion molecule-1 (VCAM-1; CD106) (181), and endothelial-leukocyte adhesion molecule 1 (ELAM-1; CD62) (182, 183).

Secondly, ET-1 activates meningeal Mast Cells (MCs) which, upon degranulation, release inflammatory cytokines, playing a contributory role in disrupting BBB (184).

Thirdly, being a powerful vasoconstrictor, it induces long-term blood flow occlusion to the area affected by ischemic insult, thus further fanning neuronal damage, ROS production, and cytokine release (185).

Contrary to this, NO is a minor biological radical which is produced by nitric oxide synthase (NOS) enzyme family from L-arginine (186). NOS have three discrete anatomical sources: neuronal NOS (nNOS), inducible NOS(iNOS), and endothelial NOS (eNOS) (187–189). Of these, eNOS have a regulatory role in BMECs (190). Normally, eNOS triggers vasodilation, inhibits platelet aggregation, and revitalizes blood flow to brain tissues; in short, they provide neuroprotective effects (191). This is the other way round in neuroinflammatory cascade where excessive ET-1 production suppresses eNOS expression and further microvascular contraction hinders blood supply to the ischemic site (192).

**Figure 7:**
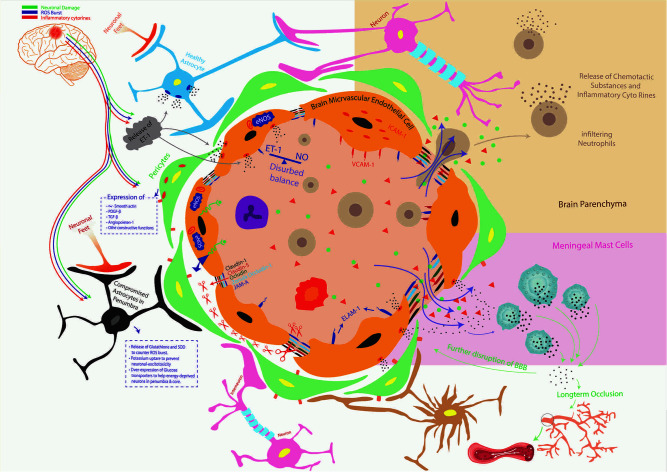
Ischemic stroke and orderly compromisation of BBB. The brain microvascular endothelial cells (BMECs), Astrocytes, Pericytes, and Microglial cells make BBB. Compromised BBB integrity is the striking preliminary feature of the neuroinflammatory-triangle during the hypoxic pathophysiological state, where astrocytes in penumbra, pericytes, and BMECs start releasing ET-1 (disturbed balance of ET-1 and NO). This leads to the upsurge in the expression of a variety of adhesion molecules in/on BMECs such as ICAM-1, VCAM-1, and ELAM-1 to facilitate trans-endothelial migration of leukocytes. ET-1 also upregulates the expression and release of matrix metalloproteinases (MMP) from BMECs which lyse inter-endothelial connecting proteins such as Claudin 1, Claudin 5, Ocludin, Zona ocludin-1, junctional adhesion molecule-A (JAM-A), and others. This compromises the barrier’s integrity, otherwise tightly maintained by BMECs, thus allowing the release of inflammatory cytokines, infiltration of systemic immune cells, and fluid escape (brain edema). Inflammatory cytokines stimulate meningeal Mast Cells to release more inflammatory mediators to further the BBB shattering and causing long-term occlusion of blood to already starved brain tissue. Along with BMECs, the functionally compromised Pericytes and Astrocytes in penumbra put their respective neurodegenerative part (crossed red-mark in dotted boxes). The population of infiltrating immune cells and their pro-inflammatory secretions proceeds a vicious neuroinflammatory circle that only aggravates brain edema and infarct volume.

In this hyper-inflammatory state, BMECs also synthesize enhanced matrix metalloprotein-2 (MMP-2) in response to inflammatory signals (**Figure 7**) (193) which digest proteins (claudin-1, claudin-5, occludin, and zonula occludens-1) (194) responsible for maintaining tight junctions between endothelial cells, further compromising the BBB integrity (195).

An additional key component of the BBB is astrocyte, which has an inverse relation with neuroinflammatory damage. Following ischemic damage, astrocytes offer recovery back into neurons because they are more resistant to glucose-oxygen deprivation mechanisms (196). Astrocytes release glutathione and SOD in response to ROS burst to reduce oxidative damage and neuronal mortality (197). Apart from this, they enhance potassium uptake to prevent excitotoxicity of neurons following post-stroke extracellular surge in the potassium levels (198). Also, astrocytes overly express glucose transporters to keep the supply of glucose to energy- stressed/dying neurons (199). This is evident by various studies which have found high levels of neuroprotective ethyl pyruvate (a derivative of the energy substrate pyruvate) only when astrocytes proximal to stroked area remain functional (200).

However, impaired astrocytes amplify neuronal damage (201). A miscalculated interactive cascade of ROS, increased pro-inflammatory cytokine production, and downregulation of anti-inflammatory cytokines along with disrupted BBB play decisive roles in astrocyte functions (202–204). The third component of BBB is pericytes which have multifaceted roles in the brain following ischemic injury.

Brain injury causes increased expression of adhesion molecules (ICAM-1 and VCAM-1) on pericytes in response to inflammatory cytokines (205, 206). In addition to this, various other molecules are overly expressed on pericytes including α-smooth muscle actin, Platelet-derived growth factor subunit B (PDGF-β), transforming growth factor-β (TGF-β), and andangiopoieten-1 (207–210). Pericytes have a constructive function against inflammatory responses, of these some include contractile maneuvers with endothelial smooth muscles, immune and phagocytic roles, migration to endothelial cells to provide supportive functions, angiogenic support, and stem cell functions (211–216). However, once the BBB losses integrity as a result of neuroinflammatory chain reaction, these supportive and angiogenic functions of astrocytes are compromised severely (217). The fourth component of the BBB, i.e., microglial cells, has been elaborated on above.

## Conclusions

Non-availability of clinically reliable therapeutic interventions for limiting stroke-related morbidities and mortalities puts the significance of clinical trials into question. This suggests that such efforts are not specifically targeted enough or that the targeted mechanisms differed between species, as most of the mechanistic studies are carried out in small animal models of stroke. Contemporary trends in stroke studies are heading towards unearthing intricate intracellular signaling pathways which are involved in neuroinflammation and stroke damage. Establishing a significant link between these pathways can only help explore potential targets for finding therapeutic interventions. The neuroinflammatory triangle entails diverse links which are central to stroke-related damage. Further reconnoitering this neuroinflammatory triangle might be rewarding and aid in finding and clinically translating novel therapeutic targets.

## Author Contributions

MK planned the study and undertook critical scrutiny of the manuscript to highlight and remove discrepancies or conflict of concepts. ZS wrote the manuscript. CA undertook review with particular focus on removing technical mistakes. AZ undertook multiple reviews to give shape to the final draft of the manuscript. DH critically reviewed the manuscript and provided input. SM contributed to manuscript writing and critically reviewed. All authors contributed to the article and approved the submitted version.

## Conflict of Interest

The authors declare that the research was conducted in the absence of any commercial or financial relationships that could be construed as a potential conflict of interest.

## Publisher’s Note

All claims expressed in this article are solely those of the authors and do not necessarily represent those of their affiliated organizations, or those of the publisher, the editors and the reviewers. Any product that may be evaluated in this article, or claim that may be made by its manufacturer, is not guaranteed or endorsed by the publisher.
